# Transition Metal Carbide Core/Shell Nanoparticles by Ultra-Short Laser Ablation in Liquid

**DOI:** 10.3390/nano10010145

**Published:** 2020-01-14

**Authors:** Angela De Bonis, Mariangela Curcio, Antonio Santagata, Agostino Galasso, Roberto Teghil

**Affiliations:** 1 Dipartimento di Scienze, Università della Basilicata, Viale dell’Ateneo Lucano, 10-85100 Potenza, Italy; mariangela.curcio@unibas.it (M.C.); agostino.galasso@unibas.it (A.G.); 2 CNR-ISM, Contrada Santa Loja, 85050 Tito Scalo (PZ), Italy; antonio.santagata@cnr.it

**Keywords:** transition metal carbide, ultrashort laser ablation in liquid, solvent degradation, core/shell nanostructures

## Abstract

Transition metal carbide nanoparticles are a class of technological interesting materials with a wide range of applications. Among metal carbides, tantalum carbides have good compatibility with the biological environment while molybdenum carbides are used as catalyst in electrochemical reactions. Laser ablation of bulk transition metal targets in some liquids is here reported and laser ablation in organic solvents is used as simple synthetic strategy for the production of carbide nanostructures. Herein, the nanoparticles produced by ultra-short laser ablation of tantalum and molybdenum in water, acetone, ethanol and toluene have been characterized by TEM, XRD and XPS analysis. The combined effect of metal and solvent chemical and physical properties on the composition of the nanomaterials obtained has been pointed out. In particular, the different reactivity of Ta and Mo with respect to oxidizing species determines the composition of particles obtained in water, on the other hand the organic solvents decomposition allows to obtain transition metal carbide (TMC) nanoparticles. The observed carbonaceous shell formed on TMC allows to protect the particle’s carbidic core and to improve and tailor the applications of these nanomaterials.

## 1. Introduction

Transition metal carbides (TMC) have high melting point and good mechanical properties, usually. Moreover, TMC nanoparticles (NPs) have outstanding performances in different applications ranging from energy storage and conversion [1] to catalysis [2,3,4]. In particular molybdenum carbides have attracted great attention in electrocatalys applications for hydrogen evolution reactions and for biomass conversion [2] as substitute for Pt based catalysts, due to Mo d-electron structure similar to Pt [5] and tantalum carbide has been used as structural reinforcement in hydrogel composites for tissue engineering applications, due to its non toxicity in biological environment [6]. The most commonly used method for the preparation of TMC is the carbonization of metal or metal oxide, this synthetic route requires long time and temperature over 1200 °C, usually [1]. Laser ablation in liquid (LAL) has been proposed as a valid option for the green synthesis of metallic, oxide and carbide NPs [7,8]. During the ablation, molecules of the surrounding liquid can decompose and react with species that are present in the laser induced hot plasma. Using organic solvents, carbon atoms and clusters can easily dissolve into transition metal nanoparticles allowing the formation of metal carbide structures. Moreover, LAL technique easily allows to synthesize nanostructrures with graphitic shells that can protect the core and improve the NPs durability [9,10]. However, despite the simple experimental set up that can be used, physical and chemical processes involved in the ablation and in particles formation and growth, could be very complex [8,11]. Therefore, complete knowledge of the ablation mechanism and the physic-chemical properties both of target materials both of solvents should be required to prepare nanomaterials with the desired composition.

Laser ablation mechanism is strongly affected by laser pulse duration. Femtosecond laser interaction with a metal target immersed in liquid induces the metal ablation by phase explosion mechanism [12]. The laser induced hot plasma allows the vaporization of the surrounding liquid that expands generating a cavitation bubble (CB). The CB grows, reaching a maximum radius that depends, mainly on the energy released on the solid surface by laser pulse. Also solvent physical properties (viscosity, density, vapor pressure…) affect the CB dynamics [13]. During the plasma cooling down, the nucleation and growth of nanoaggregates take place. Finally, hot NPs are transferred in the solvent, where they rapidly cool down [13]. With ns pulses, the absorption of the trailing part of the laser pulse by plasma plume allows to the formation of larger CB, with the formation of larger, highly crystalline nanoparticles. The ablation of a multi-elemental target by short laser source can get to the non-congruent vaporization of the ejected droplets, whereas the use of an ultrashort laser pulse, allows to retain the target stoichiometry [14].

Many authors have studied the effect of the confining liquid both on the properties of the nanostructures obtained by LAL [15,16,17], both on the ablation process [13,18]. Recent efforts have proposed that the reactions occurring between species present in CB play a key role in determining composition and structure of the obtained NPs [19] and minor process of slow growth and surface modification can take place in the room temperature solvent. Composition of nanostructures obtained by LAL can be related to the chemistry of the solvent whereas atmospheric gases, such as molecular oxygen or CO_2_ dissolved in liquid, should have effects on particles ageing modification [20]. On the other hand, liquid physical properties such as acoustic impedance can affect ablation rate as has been recently proposed by Baruah et al. [18], which have studied the role of the confining solvent during the nanosecond ablation of Cu in water and alcohols.

The nanosecond ablation in organic solvents has been proposed as a simple route for the nanoparticles synthesis of TMCs [17,19,21,22,23]. Femtosecond laser ablation in liquid of tantalum and molybdenum is here reported for the first time, to the best of our knowledge. Tantalum and molybdenum carbide (Ta_x_C_y_ and Mo_x_C_y_) nanoparticles have been obtained and characterized by microscopic and spectroscopic techniques. We discuss our results considering the different physic-chemical properties both of the two metals and of confining liquids used in LAL experiments.

## 2. Materials and Methods 

Ta and Mo metallic targets were purchased from Goodfellow with a purity over 99.99%. Each metal target was ablated in the selected solvent (water (Bi-distilled MilliQ) and acetone, ethanol and toluene from Aldrich) for 10 min using a Ti: sapphire laser source (Spitfire, 1 kHz, 800 nm, 100 fs, 3 mJ for each ablation). In order to minimize non linear optical effects, laser source was focused perpendicularly on the target surface by means of a 50 mm focal plane lens and the liquid column above the target was fixed at about 2 cm. The focus was just on the metal target surface with a fluence of 2 J/cm^2^.

The products were centrifuged (6000 RPM, 30 min) and dried for XRD and XPS measurements, performed by using X-Perth-Pro Philips and LH-Leybold X1 instrument, respectively. The acquired XPS spectra were analyzed by a curve-fitting program, Googly that allows the simultaneous fitting of peaks in the form of a Voigt function and their associated background in a wide energy range [24]. Colloidal solutions have been drop casted onto holey carbon copper grids for TEM characterizations (FEI-TECNAI 200 kV). More than 500 NPs have been counted to obtain the NPs size histograms.

## 3. Results

### 3.1. Tantalum

**Figure 1:**
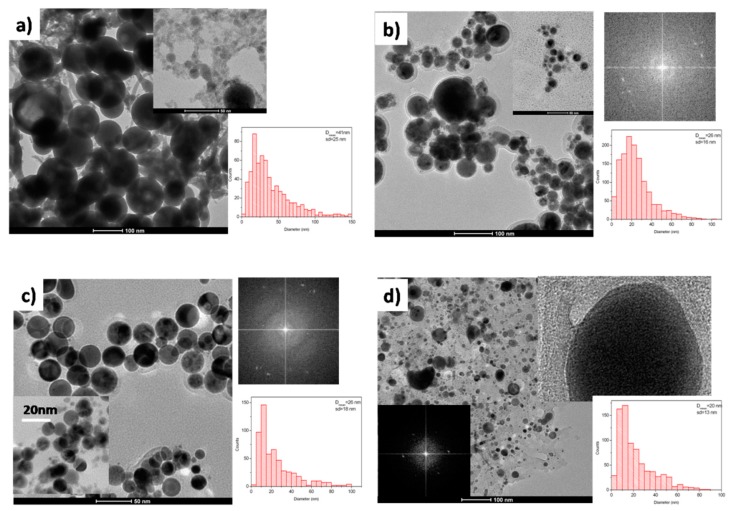
TEM images and particles size distributions of nanoparticles obtained by laser ablation of Ta in water (**a**), ethanol (**b**), acetone (**c**) and toluene (**d**).

In Figure 1a–d, are reported TEM images of nanoparticles obtained by laser ablation of tantalum in water, ethanol, acetone and toluene, respectively. By using water as confining liquid, spherical nanoparticles, with a mean diameter of about 40 nm, together with smaller ones with a diameter <10 nm and layered structures have been obtained (Figure 1a). Smaller particles are aggregated and strongly connected, whereas the presence of layered structures can be related to an anisotropic growth of tantalum oxide, as already reported for manganese oxide obtained by laser ablation in water [25]. 

**Figure 2:**
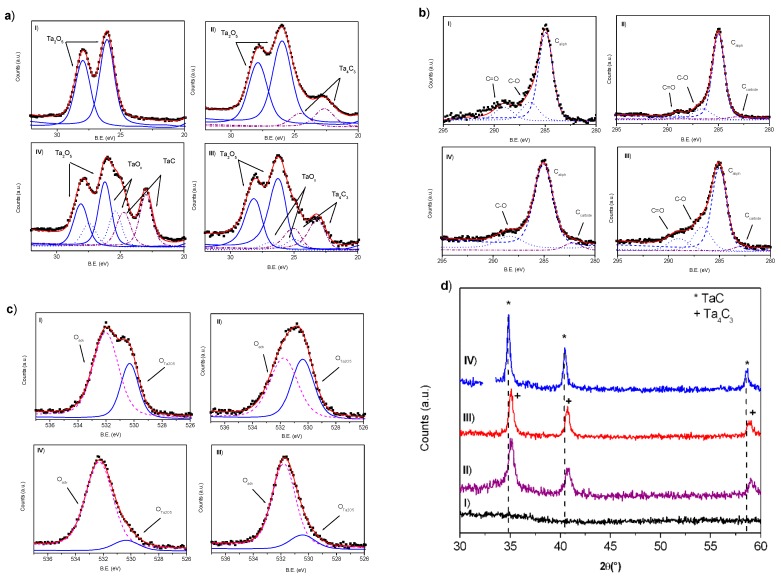
(**a**) XPS Ta 4f region spectra; (**b**) XPS C1s region spectra; (**c**) XPS O1s region spectra and (**d**) XRD spectra of nanoparticles obtained by laser ablation of Ta in water (I), ethanol (II), acetone (III) and toluene (IV). In XPS spectra blue lines are referred to Ta_2_O_5_, blue dotted lines to TaO_x_ and purple dash-dotted lines to Ta_x_C_y_ species, respectively.

Unexpected, none crystalline domain has been observed for nanoparticles obtained in water and their amorphous state has been confirmed by XRD analysis (Figure 2d(I)). XPS spectrum of nanomaterials obtained by tantalum ablation in water is reported in Figure 2. Ta 4f region (Figure 2a(I)) consists of a doublet from the spin orbital splitting of 4f electrons in 4f_7/2_ and 4f_5/2_ signals. The Ta 4f_7/2_ peak presents a single component centered at 25.8 eV, a B.E. that can be referred to Ta_2_O_5_ species [26]. None evidence of metallic tantalum has been observed. The evaluation of the position (B.E. = 530.3 eV) and intensity of O1s peak (Figure 2c(I)) confirms this assignment and C1s spectral feature is typical of adventitious carbon (Figure 2b(I)). The complete oxidation of the tantalum NPs obtained by LAL in water is not surprising, considering the high affinity of tantalum with respect to oxygen and the very negative formation enthalpy of Ta_2_O_5_ (∆H= −2046 kJ/mol) [27]. 

With the aim to obtain metal carbide NPs, some organic solvents with different C/O and C/H ratio have been used as confining liquid in laser ablation experiments. Ethanol and acetone have been selected since it is possible to investigate the effect of different oxygen chemistry on the composition of the produced NPs, whereas toluene has the higher C/H ratio among the considered liquids and different chemical reactivity due to the presence of an aromatic ring. In Figure 1b NPs obtained by tantalum ablation in ethanol are reported. Spherical particles with a lighter amorphous shell can be observed. By size distribution of more than 1000 particles a mean size of 26 nm has been evaluated. These particles show many crystalline domains where lattice distances of 0.28, 0.25 and 0.24 nm have been evaluated by 2D-FFT. The lattice distance of 0.24 nm can been assigned to (220) planes of cubic Ta_2_O species [28,29] and that of 0.25 nm to the non-stoichiometric Ta_4_C_3_ (111) distance [30]. In XRD spectrum of NPs obtained by tantalum ablation in ethanol (Figure 2d(II)) the diffraction signals at 35.14°, 40.80° and 58.90° are assigned to (111), (200) and (220) planes of Ta_4_C_3_ (PDF: 03-065-3191), respectively. The crystallite size of 12 nm, evaluated by Scherrer method considering the Ta_4_C_3_ (111) peak, confirms that the obtained NPs are polycrystalline. The Ta4f_7/2_ peak in XPS spectrum (Figure 2a(II)) has been resolved in two components centered at B.E. of 25.9 and 22.6 eV, that can be assigned to Ta_2_O_5_ and tantalum carbide species [31,32], respectively. In C1s region (Figure 2b(II)) the low intensity component at B.E. 282.1 eV has been assigned to carbon bonded to tantalum and considering the C/Ta area ratio of 0.74, the Ta_4_C_3_ stoichiometry has been evaluated. By XPS analysis, surface of NPs obtained in ethanol is composed by about 18% of tantalum bonded to carbon and 82% of tantalum oxide as Ta_2_O_5_.

**Figure 3:**
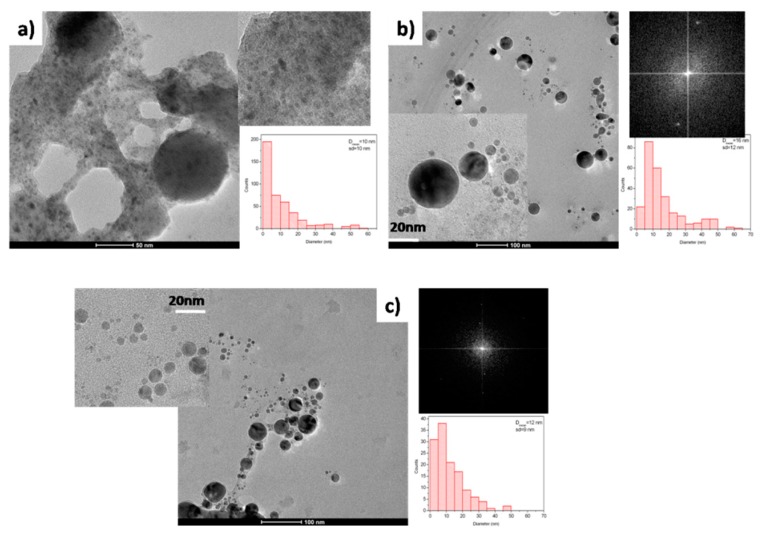
TEM images and particles size distributions of nanoparticles obtained by laser ablation of Mo in water (**a**), acetone (**b**) and toluene (**c**).

Very similar results have been obtained by laser ablation of tantalum in acetone. Spherical NPs with a lighter amorphous shell and with a mean diameter of 26 nm can be observed in Figure 1c. Crystalline domains are visible and lattice distances of 0.28 and 0.24 nm have been evaluated. XRD spectrum (Figure 3d(III)) suggests the presence of crystalline Ta_4_C_3_ with crystalline domains of about 14 nm. Irrespective from these similarities, nanoparticles obtained by laser ablation in acetone and ethanol present some differences that can be observed by XPS analysis. In fact, in the Ta4f_7/2_ signal of acetone mediated particles (Figure 2a(III)) presents a shoulder at lower BE with respect to the Ta_2_O_5_ component. This shoulder, centered at 24.9 eV can be related to suboxide tantalum species [33]. The component at 23.1 eV confirms the presence of tantalum carbide, with a Ta_4_C_3_ stoichiometry. The amount of tantalum bonded to carbon has been evaluated of about 26%. The higher amount of tantalum carbide and the presence of sub-stoichiometric tantalum oxide species suggest that the different chemistry of alcoholic and chetonic oxygen in ethanol and acetone, respectively, can play a role in determining the composition of the NPs obtained during the ablation process. In particular, the oxidizing effect seems to be more effective for ethanol than for acetone.

Nanostructures obtained by laser ablation in toluene (Figure 1d) present large crystalline domains where a lattice distance of 0.26 nm has been measured, a distance that has been related to the spacing of (111) planes of cubic TaC [34]. The particles show a core/shell structure where the typical Onion Like Carbon (OLC) phase can be observed. Particles with a mean diameter of 20 nm have been evaluated by size distribution, also if some larger ones are present. XRD spectrum (Figure 2d(IV)) presents signals at 34.84°, 40.44° and 58.53°, confirming the formation of stoichiometric tantalum carbide (TaC) (PDF: 01-089-2870) with crystalline domains of about 21 nm. XPS analysis (Figure 2a–c(IV)) allows to evaluate that the surface of the obtained NPs is composed by about 40% of tantalum bonded to carbon. The intensity ratio of Ta4f_7/2_ signal at 23.51 eV and C1s at 282.8 eV confirms the TaC stoichiometry.

### 3.2. Molybdenum

Molybdenum has lower affinity with respect to oxygen compared to tantalum, with enthalpy of formation for MoO_3_ and MoO_2_ of −741 kJ/mol and −587 kJ/mol, respectively [27]. In TEM image of material obtained by laser ablation of a molybdenum target in water many small particles (<10 nm) are visible (Figure 3a), together with some particles larger than 50 nm.

**Figure 4:**
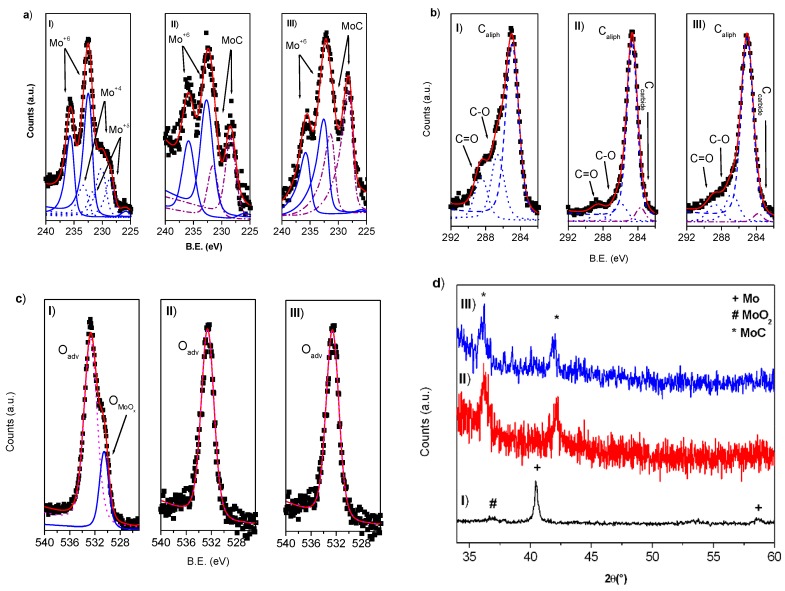
(**a**) XPS Mo 3d region spectra; (**b**) XPS C1s region spectra; (**c**) XPS O1s region spectra and (**d**) XRD spectra of nanoparticles obtained by laser ablation of Mo in water (I), acetone (II) and toluene (III). In XPS spectra blue lines are referred to MoO_3_, blue dotted lines to Mo^+2^ and Mo^+δ^ and purple dash-dotted lines to MoC species, respectively.

XPS analysis allows to observe the complex surface chemistry of nanoparticles obtained in water. In fact, the Mo3d_5/2_ peak (Figure 4a(I)) is composed by three contributions: a larger one, centered at B.E. of 232.5 eV, and two other ones at 230.0 and 228.6 eV, assigned to Mo^+6^, Mo^+4^ and Mo^+δ^ species, respectively [35]. O1s peak (Figure 4c(I)) has been resolved with two components at 530.5 end 532.5 eV, assigned to molybdenum oxides and to oxygen bonded to contamination carbon, respectively. In XRD spectrum (Figure 4d(I)) peaks at 40.5° and 58.6° are referred to (110) and (200) crystal planes of cubic Mo (PDF 01-089-4896) and the low intensity component at 37.0° has be assigned to MoO_2_ species (PDF 03-065-1273).

In Figure 3 particles obtained by femtosecond laser ablation of molybdenum in acetone (b) and toluene (c) are reported. Spherical particles with a diameter of 16 and 12 nm have been obtained, respectively. Particles obtained by laser ablation both in acetone and in toluene present nanocrystalline domains with lattice distance of 0.25 and 0.22 nm, suggesting the presence of cubic MoC crystal domains [36]. The usual OLC structure is clearly visible for particles obtained in toluene. XRD analysis (Figure 4d(II–III)) allows to observe signals at 36.38° and 42.27°confirming the formation of MoC crystalline domains (PDF: 03-065-0280). None evidence of metallic molybdenum and molybdenum oxides is present.

XPS analysis shows that the Mo3d_5/2_ peaks for materials obtained in acetone and toluene (Figure 4a(II–III)) are very similar and present two contributions centered at 232.7 and 228.3 eV, that can be related to Mo^+6^ and molybdenum carbide species [37], respectively. The Mo/C ratio calculated considering the 283.4 eV peak in the C1s signals (Figure 2c(II–III)) confirms the MoC stoichiometry. It was possible to evaluate a molybdenum carbide percentage of about 40% and 60% for the ablation in acetone and toluene, respectively. The evaluated carbide amount is sensitively higher with respect to the carbide percentage measured for nanoparticles synthesized using metallic tantalum target in the same experimental conditions.

## 4. Discussion

To understand the final composition of the NPs obtained by laser ablation in liquid, it is necessary to consider the mechanism of ultra-short laser interaction with a solid target confined in a liquid medium. It has been reported that femtosecond ablation follows a phase explosion mechanism [38], with the formation of a very dense plasma, consisting of atoms, small clusters and NPs, whose composition is dominated by the target one. The hot laser induced plasma starts the decomposition of the surrounding liquid with the formation of ions, radicals and atoms that react with plasma species. The expansion and cooling down of the plasma follow with the vaporization of the surrounding liquid. The vapor layer confines the laser induced plasma and generates a CB, with dynamics that comprise expansion and collapsing stages, repeated several times [13]. During the plasma cooling down, nucleation, growth and coalescence of NPs can take place. Particles start to cool down in the CB and then the hot NPs, ejected during the CB collapsing stage, quickly cool down at room temperature in the solvent, due to the lower thermal capacity of liquid with respect to gases. Minor processes of slow growth or chemical reactions with the surrounding liquid or gasses can occur at this stage [20]. Recent efforts have demonstrated that the reactions occurring between species in CB play a key role in determining composition and structure of the obtained NPs [16]. By using water as confining liquid, in the laser induced CB, vapor water, OH^−^ and oxygen radicals are present. Due to the high concentration of elemental metal species and the high temperature of the plasma zone, the formation of metal nanoparticles or metal oxide ones can be related to the metal redox potential and to the enthalpy of formation of the metal oxides [7]. The high affinity of Ta with respect to oxygen is confirmed by the formation of Ta_2_O_5_ nanoparticles, with none evidence of metallic Ta, during the ablation of the metal Ta target in water. On the other hand, the ablation of molybdenum, that has higher redox potential [27] and higher enthalpy of formation for its oxides with respect to tantalum, allows the formation of nanoparticles that are non completely oxidized, as observed by XRD measurement. This behavior is confirmed by the results obtained by laser ablation in water of metals with high redox potential such as Pt and Pd, where metallic NPs with a lighter oxide shell have been obtained [39,40,41,42].

Laser ablation in organic solvents of transition metals is a synthetic route that can be used for the preparation of metal carbidic structures. Transition metal elements with a high number of unfilled d-orbitals have high affinity to carbon, due to the possibility to entrap carbon atoms in the metal vacancies and to get to the formation of metal carbon bonds [19]. Usually, these transition metal carbides are thermodynamically stable as can be observed considering their enthalpy of formation [43]. Acetone and ethanol have similar physical properties (density, thermal capacity and polarity) and C/O ratio, but they have different chemical properties due to the presence of a chetonic and an alcoholic oxygen, respectively. The amount of carbon and hydrogen species, formed during acetone and ethanol degradation, is higher with respect to oxygen, reflecting the solvent molecular formula and the bond energies of the molecules [18]. The interaction of metals and carbon atoms in the CB at high temperature can induce the formation of crystalline metal carbides with a composition and percentage that reflect carbon affinity of the metal and metal carbide thermodynamic stability. In fact Ta, Mo, Ti [9], Co [22], W [44], Nb [23] carbides have been obtained by laser ablation in acetone and/oralcohols, on the other hand, the ablation of metals, such as Fe [45] Pd [40] and Cu [18], in acetone and/or alcohols gets to the formation of metallic and/or oxide particles. During their thermal degradation, acetone and ethanol can follow different pathways, getting to the formation of products with dissimilar chemical properties. In fact, acetone decomposes producing reductive gas as CO, and C_2_H_6_ [19] whereas ethanol decomposes forming species such as ethene, methane, but also H_2_O and O_2_ [46]. Considering the formation of water, a higher amount of oxidized species can be expected when the ablation is performed in ethanol with respect to acetone, as confirmed by XPS analysis of species obtained by tantalum ablation. As a result, in order to obtain metal carbide nanostructures acetone should be preferred to ethanol. Outside the CB, the dissolved carbon precipitates on the NPs surface, due to particles rapid quenching. Transition metal ablation in acetone by nanosecond laser source allows to obtain core/shell nanostructures with crystalline carbidic core and a graphitic shell, where up to 28 carbon layers have been measured [19,22]. However, due to the inessential overlap between laser pulse and plasma plume and to the short electron cooling time of the metal target and surrounding liquid, the formation of graphitic shells during ultra-short laser ablation of metals in acetone is greatly reduced [10] and the NPs embedded in an amorphous carbon matrix are usually obtained, even considering the ablation metals with good catalytic ability such as Pd, Ti, Ta and Mo [9,40]. Due to the capping effect of the organic molecules or the organic byproducts, the growth and coalescence of particles outside the CB is strongly limited and NPs produced by laser ablation in acetone or ethanol are smaller with respect to particles synthesized in water [19]. With respect to tantalum and molybdenum carbides obtained by nanosecond laser source [19,21,36] the use of an ultrashort laser source allowed to obtain smaller nanoparticles, where a single carbidic phase is present. Moreover, it has been reported that the laser ablation of a tantalum target in ethanol gets to the formation of TaxO@Ta_2_O_5_ particles, due to the water molecules dissolution around the hot plasma plume [28], whereas the shorter lifetime of fs-induce plasma and CB allows to obtain a carbidic core.

Toluene is a carbon rich solvent with higher density with respect to acetone, but lower polarity and thermal capacity. Due to toluene decomposition in the CB, a high amount of carbon atoms can be expected. Moreover, the formation of graphitic shells can be related to the pyrolysis of the organic molecule [47,48] due to the high temperature reached in the CB site. However, by using ns laser source the toluene pyrolysis can be due also to the interaction with the trailing laser pulse and Cristoforetti et al. [49] shown that the ablation of Pd in hydrocarbon such as n-hexane and toluene is inefficient, due to a low rate of ablation and the formation of unstable colloidal solution. This effect is strongly limited by ultra-short laser ablation and toluene is the most effective solvent for the production of OLC/metal carbide core/shell nanostructures. In fact, the hot NPs ejected during the CB collapsing step, can act as nucleation sites for toluene thermal decomposition and graphitization. Toluene low polarity [50] and the capping effect of the graphitic shell, can prevent the growth of particles and can justify their lower dimension with respect to particles obtained by laser ablation in acetone and ethanol. As a result, TaC and MoC nanocrystalline core/shell particles have been obtained. The formation of OLC structure could be beneficial lending to the NPs improved properties in terms of high specific surface area, high electrical conductivity and good tribological properties [51,52,53]

## 5. Conclusions

We have studied the ultra-short laser ablation of Ta and Mo in water and organic solvents with different chemistry with the aim to highlight the combined effect of chemical properties of targets and solvents and of the reactions that take place in the laser induced CB on nanoparticles composition. The amount of oxidized species obtained by laser ablation in water reflects the metal chemical properties and redox potential; in fact the ablation of molybdenum gets to the formation of metal/metal oxide nanoparticles, whereas Ta_2_O_5_ has been obtained by using a tantalum target. By ultrashort laser ablation in acetone and ethanol metal transition carbide particles have been obtained. However, irrespective of their similar physical properties and C/O ratio, the thermal decomposition of these solvents follows different chemical pathways and gets to the formation of species with different oxidizing ability. As a result, acetone should be preferred for the preparation of carbidic particles by ultra-short LAL technique. Due to the ultra-short laser pulse duration, acetone and ethanol graphitization is weakly effective on the surface of the hot ejected nanoparticles and OLC shells are present only by laser ablation in toluene. Moreover, considering its high C/H ratio, metal ablation in toluene gets to the formation of stoichiometric TaC and MoC species.
